# The Integral Role of Hospice Nurse Educators in a Palliative Care Education Program for Nurses in Assisted Living

**DOI:** 10.1093/geroni/igaa057.2452

**Published:** 2020-12-16

**Authors:** Debra Dobbs, Hongdao Meng, William Haley, Harleah Buck, Kathryn Hyer, Maureen Templeman, Carlyn Vogel

**Affiliations:** University of South Florida, Tampa, Florida, United States

## Abstract

Persons with dementia (PWD) are increasingly cared for in assisted living (AL) settings with an annual mortality rate of close to 20%. Palliative care (PC) for PWD in ALs can improve end-of-life care. From May, 2019 to February, 2020 a 4-week PC education in AL (PCEAL) program for nurses who provide care to PWD, facilitated by hospice nurses in Florida, was tested in a sample of nurses (N=20) in a cluster randomized trial (9 ALs, 4 treatment/5 control). We examined if PC knowledge increased from pre to post-intervention using a validated measure (Thompson, 2011). All intervention nurses (N=10) completed all four sessions of the PCEAL. While the baseline score was lower for the intervention group compared to the control group, the intervention group improved (M=2.20 to 2.37) compared to the control group (M=2.83 to 2.75) post-intervention. Two-month booster sessions indicate nurses have integrated PC care learned in the PCEAL program.

